# Supporting Unpaid Caregivers of Persons Living With Dementia: Protocol for a Pilot Feasibility Study to Explore Caregiver Outcomes and Impact of a Co-Designed Simulation–Based Psychoeducation Program in Virtual Reality

**DOI:** 10.2196/87107

**Published:** 2026-03-05

**Authors:** Mary Chiu, Sarah C Pistritto, Kristina M Kokorelias, Krystina M Clarke, Andrei Torres, Cole G Craven, Adam Dubrowski, Bill Kapralos, Erica O’Hare, Joel Sadavoy, Adriana Shnall, Michael S D Smith, Nusrat Choudhury, Amer M Burhan

**Affiliations:** 1 Research & Academics, Ontario Shores Centre for Mental Health Sciences, 700 Gordon St, Whitby, ON, L1N 5S9, Canada, +1 905-430-4055; 2 Faculty of Health Sciences, Ontario Tech University, Oshawa, ON, Canada; 3 Section of Geriatrics, Sinai Health and University Health Network, Toronto, ON, Canada; 4 Rehabilitation Sciences Institute and Department of Occupational Sciences and Occupational Therapy, Temerty Faculty of Medicine, University of Toronto, Toronto, ON, Canada; 5 KITE-University Health Network, Toronto, ON, Canada; 6 Faculty of Computer Science, Ontario Tech University, Oshawa, ON, Canada; 7 maxSIMhealth Group, Ontario Tech University, Oshawa, ON, Canada; 8 Game Development and Interactive Media, Ontario Tech University, Oshawa, ON, Canada; 9 Department of Psychiatry, Mount Sinai Hospital, Toronto, ON, Canada; 10 Department of Psychiatry, Temerty Faculty of Medicine, University of Toronto, Toronto, ON, Canada; 11 The Koschitzky Centre for Innovations in Caregiving, Baycrest Academy for Research and Education, Toronto, ON, Canada; 12 Factor Inwentash Faculty of Social Work, University of Toronto, Toronto, ON, Canada; 13 Medical Devices, National Research Council Canada, Winnipeg, ON, Canada; 14 Medical Devices, National Research Council Canada, Boucherville, ON, Canada

**Keywords:** protocol, dementia, family caregivers, resilience, empathy, virtual reality, simulation-based learning, caregiver stress, burden, experiential learning

## Abstract

**Background:**

Dementia is a global public health concern, with prevalence projected to reach 78 million individuals by 2030 and 139 million by 2050. Most persons living with dementia reside in community settings and are supported by family caregivers. As caregiving demands grow, caregivers experience significant psychosocial, emotional, and financial burden, including high rates of stress, social isolation, and depressive symptoms. Access to effective support services remains limited, highlighting the urgent need for innovative and accessible caregiver interventions.

**Objective:**

This pilot study first aims to assess the feasibility, acceptability, and tolerability of VR-SIM Carers, a virtual reality (VR)–based psychoeducational training program for family caregivers or care partners of people living with dementia. Second, it will aim to provide a preliminary evaluation of potential impact on caregiver outcomes, including caregiver competence, stress, resilience, empathy, and quality of life. The study is not designed to support causal inference regarding the effectiveness of VR-SIM Carers.

**Methods:**

A mixed methods design will be used with a sample of 30 family caregivers of people living with dementia. Participants will complete 3 immersive, self-paced VR caregiving scenarios—Managing Apathy, Crisis Response, and Refusal of Care—receiving real-time feedback from simulated characters, including clinician and person living with dementia avatars. Primary outcomes (feasibility and educational impact) include recruitment, retention, adherence, usability, acceptability, and tolerability and caregiver competence (Pearlin Caregiving Competence Scale), perceived stress (Cohen Perceived Stress Scale), resilience (Connor-Davidson Resilience Scale), and empathy (Empathy Assessment Scale). Secondary outcomes (preliminary efficacy) include caregiver quality of life (Adult Carer Quality of Life Questionnaire), caregiver burden (Burden Questionnaire), and behavioral symptoms (Neuropsychiatric Inventory, Center for Epidemiologic Studies Depression Scale Short Form) assessed at baseline, postintervention, and 1-month follow-up. Feasibility and user engagement will be evaluated via the 18-item Gaming Use Engagement and Severity Scale, Igroup Presence Questionnaire, qualitative interviews, reflection notes, and open-ended feedback. Quantitative data will be analyzed using repeated-measures ANOVA and paired 2-tailed *t* tests, while qualitative data will be analyzed using an inductive thematic coding framework. Data analyses are descriptive and exploratory only, and no causal claims regarding intervention effectiveness will be made. Consistent with CONSORT (Consolidated Standards of Reporting Trials) guidance for pilot and feasibility studies, caregiver outcomes (eg, competence, stress, resilience, empathy) are treated as exploratory.

**Results:**

The findings from this study will inform the feasibility, acceptability, and educational value of immersive VR for caregiver training, while providing preliminary evidence regarding the efficacy of VR-SIM Carers as a training tool to improve psychoeducation outcomes for family caregivers of people living with dementia and reduce caregiving burden. Data collection commenced in March 2024 with a projected end date of March 2026. As of the submission of the manuscript (December 2025), 21 participants have been enrolled. Data analysis will be completed in April 2026, and the results are expected to be published in fall 2026.

**Conclusions:**

VR-SIM Carers represents an innovative, scalable intervention designed to enhance caregiver preparedness, psychosocial outcomes, and sustainable community–based dementia care. This pilot study will provide critical evidence to guide further development and implementation of VR-based caregiver support programs.

## Introduction

### Background

Dementia is a growing global public health challenge with profound personal, societal, and economic implications. As the world’s population ages, the prevalence of dementia is projected to increase dramatically: in 2019, an estimated 55.2 million people were living with dementia worldwide—a figure expected to rise to 78 million by 2030 and 139 million by 2050 [1]. This demographic shift is mirrored in Canada, where the incidence of dementia is rising alongside the aging population. By 2030, nearly 1 million Canadians are expected to be living with dementia, and this number is projected to double by 2050 [2]. In Canada, approximately 61% of people with dementia reside at home, instead of in long-term care facilities [3]. This trend reflects both the wishes of older adults to age in place and the increasing reliance on unpaid caregivers—primarily family members, friends, and neighbors—to provide daily support [3]. In low- and middle-income countries, where formal care services are often limited, family caregiving is nearly universal; however, even in high-income countries, such as Canada, the majority of people living with dementia continue to rely on unpaid care arrangements [4].

Unpaid caregivers are the backbone of dementia care, making a critical yet often under-recognized contribution to the health system and society. In Canada alone, family caregivers or care partners provide an estimated 330 million hours of unpaid care annually, a figure projected to nearly double to 640 million hours by 2040 [5]. The economic value of this contribution is staggering; in 2016, the combined health system and out-of-pocket costs for people with dementia reached $10.4 billion, with unpaid caregiving accounting for a substantial proportion of this total [5]. By 2050, the annual hours of unpaid care provided by friends and family are expected to reach 1.4 billion—the equivalent of over 690,000 full-time jobs [2].

### Chronic Stress in Family Caregivers Associated with Higher Risk of Dementia

Caring for a person living with dementia is associated with substantial emotional, psychosocial, and financial burdens. Numerous studies have documented elevated rates of stress, anxiety, depression, and social isolation among family caregivers or care partners of people living with dementia [6,7]. A recent Canadian study found that 86% of those caring for both aging parents and their own children—sandwich generation caregivers—reported negative impacts on their health and well-being, including high levels of stress, exhaustion, and depressive symptoms [3]. Women constitute the majority of caregivers, and those who fall under the category of “sandwich generation” are particularly vulnerable to emotional distress and burnout, likely due to both cultural expectations and the hands-on nature of their caregiving roles [3,6]. Similarly, caregivers who are concurrently employed experience disproportionately high levels of stress combined with a low sense of mastery when faced with caring for mental and behavioral demands of older adults with dementia [8].

In particular, the chronic stress associated with dementia caregiving is a risk factor for increased risk of cognitive decline and dementia in caregivers themselves [7,9]. Prolonged exposure to caregiving stress is associated with elevated cortisol levels, immune dysregulation, and a greater incidence of cardiovascular disease and metabolic syndrome [9]. Furthermore, caregivers with high perceived stress are less able to engage in self-care, leading to a vicious cycle of declining health and reduced caregiving capacity [7]. Given these risks, interventions may need to consider both supporting caregivers in their roles but also addressing their own health and well-being. This is particularly crucial for family caregivers or care partners of people living with dementia who are burdened, stressed, and lacking the skills and knowledge to provide effective care.

### Evidence-Based Interventions for Family Caregivers or Care Partners of People Living with Dementia

A broad array of interventions has been developed to support family caregivers or care partners of people living with dementia, including support groups, counseling, psychoeducational programs, and respite care [10,11]. Among these, psychoeducational and multimodal structured psychotherapy have shown promise in reducing caregiver burden, stress, and depressive symptoms while enhancing caregiver competence and emotional resilience [7,12]. Structured programs that combine disease-specific knowledge, practical caregiving skills, psychotherapy, and emotional support have been shown to improve caregiver competence, reduce anxiety, and foster effective care strategies [7,8,13]. Multimodal interventions that integrate education, communication skills training, and problem-solving techniques offer a holistic approach to managing the complex demands of dementia care [12]. Support groups, whether in-person or online, foster a sense of community and shared understanding, reducing social isolation and promoting psychological well-being [11,14].

Despite their effectiveness, many traditional interventions are limited by in-person delivery models that can be difficult for caregivers to access regularly, especially those balancing work and/or living in rural and underserved communities. Barriers, such as time constraints, transportation challenges, and stigma, further impede engagement with support services. There is a need for innovative, accessible, and evidence-based interventions that can be delivered flexibly and at scale, particularly for caregivers who are most burdened, stressed, and at risk of declining health.

### Opportunity: Tech-Enabled Caregiver Support and Intervention

Simulation-based learning has long been a gold standard in professional health care education, offering a structured and psychologically safe environment to practice clinical skills, improve communication, and develop emotional intelligence [15]. Technologies, such as virtual reality (VR), can support the delivery of immersive simulation–based psychoeducation in an accessible, scalable, and engaging format. Recent studies have shown that VR-based training for both professional and family caregivers of people living with dementia can improve knowledge, compassion, and behavioral competence, in addition to supporting meaningful changes in caregiving practice [16]. Importantly, VR-based interventions can be designed to be self-paced and are potentially accessible from home or community settings, in urban or rural settings, offering flexibility and autonomy that are particularly valuable for caregivers juggling multiple responsibilities [16]. Interventions that include options for caregiver reflection, expert guidance, and resource linkage further enhance user experience and implementation potential [17-19]. Although evidence on effectiveness is still emerging, the preliminary findings are promising; immersive VR simulations can support empathy-building, skill acquisition, and confidence in care, all of which are central to caregiver preparedness, confidence, and well-being [19,20].

VR simulations not only enhance skill development but may also promote empathy by allowing caregivers to hear feedback from the character representing people living with dementia. When coupled with opportunities for guided reflection and clinical debrief, this form of intervention holds strong potential to shift caregiving behavior and improve caregiver outcomes. Moreover, as immersive technologies become more affordable and user-friendly, they represent a promising avenue for caregivers to access simulation-based, experiential learning and practice that is skills-based and emotionally engaging. However, few initiatives to date have rigorously explored the feasibility and acceptability of delivering simulation-based psychoeducation for family caregivers or care partners of people living with dementia through VR or evaluated its potential impact on caregivers’ psychosocial and mental health outcomes.

### VR-SIM Carers: Program at a Glance

The VR-SIM Carers Minimally Viable Prototype (MVP) is a self-paced, VR-based training tool designed to help family caregivers or care partners of people living with dementia develop practical caregiving strategies through immersive simulation–based learning. Developed using a co-design approach with input from older adults, caregivers, clinicians, simulation educators, and computer scientists, the MVP is accessible via commercially available headsets and aims to replicate real-world caregiving challenges, allowing users to learn and practice in a safe and controlled environment. The current prototype includes 3 interactive VR scenarios focused on challenging situations commonly encountered by family caregivers or care partners of people living with dementia. These scenarios are “high-fidelity,” meaning they closely replicate real-world caregiver situations with a high degree of realism. The content was inspired by real-life caregiver experiences discussed in a clinical setting during the Reitman Centre CARERS Program [8,21]. The 3 scenarios included in the MVP—Managing Apathy, Crisis Response, and Refusal of Care—were chosen through a priority setting survey completed by 16 knowledge users, family caregivers or care partners of people living with dementia and health or social providers and clinicians. Each scenario takes approximately 10 minutes to complete and may be repeated as needed. The further functionality of the MVP is detailed in the *Methods* section.

Psychoeducation is a therapeutic approach focused on educating individuals about their mental health conditions, treatments, and coping strategies, empowering them with essential knowledge for effective management. The development of our VR-based psychoeducational program adheres to the VR-CORE framework described by Birckhead et al [22], a set of guidelines and recommendations for the development and testing of VR treatments through 3 phases (VR1, VR2, and VR3), akin to the U.S. Food and Drug Administration’s Phase I-III framework for pharmaceuticals but adapted for VR. The objectives of this pilot study (see below) fit within the VR2 phase, which emphasizes early testing to assess the feasibility, acceptability, and tolerability of the VR intervention, along with its potential clinical efficacy. End user feedback will be obtained in order to explore the program’s readiness and identify implementation challenges. Our program targets outcomes, such as enhancing caregiver relationships with people living with dementia, boosting caregiver competency and resilience, and reducing caregiver stress.

### Research Objectives

The primary objective of the pilot study is to evaluate the feasibility, acceptability, and tolerability of immersive VR technology as a tool to deliver psychoeducation content to family caregivers or care partners of people living with dementia, as well as its readiness for implementation.

The secondary objective is to examine the initial efficacy of VR-based training in improving psychosocial outcomes of family caregivers or care partners of people living with dementia.

## Methods

### Methodological Approaches and Timeline

The pilot study will use a mixed methods approach to assess feasibility, acceptability, and tolerability of the VR-SIM Carers MVP and collect preliminary data on caregiver outcomes. Quantitative outcome measures and assessment tools will be administered at 3 time points: baseline, postintervention, and 1-month postintervention, as illustrated in Table 1. For both objectives, qualitative data will be collected through semistructured interviews with participants (Supplementary file B in Multimedia Appendix 1). In addition, multiple sources of qualitative data—1) field notes taken at the week 2 or 3 check-in phone call, 2) participants’ written reflection notes, and 3) open text responses to survey questions at 1-month follow-up—will be analyzed to assess feasibility, acceptability, and tolerability of VR-SIM Carers from the perspectives of the caregivers. Data collection began in March 2025, and we anticipate all data analyses to be completed by April 2026. Reporting preliminary efficacy will follow the CONSORT (Consolidated Standards of Reporting Trials) extension for pilot and feasibility trials; a CONSORT flow diagram will be included to report numbers screened, eligible, enrolled, allocated, assessed at each time point, and retained, with the reasons for exclusions and losses to follow-up.

**Table 1.** Self-completed assessment scales and respective completion time points.

Scale or survey	Baseline (week 1)	Post intervention (week 4)	1-month follow-up
Exploratory outcomes and descriptors			
Caregiver competence	✓	✓	✓
Connor-Davidson Resilience Scale	✓	✓	✓
Empathy Assessment Scale	✓	✓	✓
Perceived Stress Scale	✓	✓	✓
Neuropsychiatric Inventory	✓	✓	✓
Quality of life scale	✓	✓	✓
Baseline descriptors only			
Center for Epidemiological Studies Depression Scale	✓		
The Burden Questionnaire	✓		
Demographic survey		✓	
Feasibility and tolerability			
18-item Gaming Use Engagement and Severity Scale			✓
Igroup Presence Questionnaire			✓
Qualitative responses (4 questions)			✓

Attrition, missing surveys, and incomplete scale data will be treated as feasibility outcomes and reported transparently at each assessment time point. Quantitative analyses will primarily use available case summaries. Where appropriate, simple descriptive sensitivity analyses (eg, last observation carried forward or comparison of complete vs incomplete cases) may be conducted to assess the robustness of the observed trends. No formal imputation or model-based inference is planned, consistent with CONSORT recommendations for pilot and feasibility studies. Standardized assessment tools for depressive symptoms (Center for Epidemiological Studies Depression Scale) and caregiver burden (the Burden Questionnaire) are completed at baseline only and are intended as descriptive sample characteristics and contextual variables rather than outcomes or indicators of intervention effectiveness.

### Intervention Description

As aforementioned, the VR-SIM Carers MVP is a self-paced, VR-based training tool designed to support family caregivers and care partners of people living with dementia in developing practical caregiving strategies through immersive simulation–based learning. The MVP consists of three key components:

1. Functional system menu: Upon entering the VR-SIM Carers platform, users are greeted with a brief narration describing how to select and start a scenario. Users may select from 1 of 3 scenarios.
2. Three high-fidelity VR scenarios: They allow for simulation-based learning and practice. Users choose from responses presented to them to move forward in the scenarios, while receiving real-time guidance and feedback from 2 nonplayer characters in the VR scenario: a virtual clinician (Figure S1 in Multimedia Appendix 1) and a simulated person living with dementia (a virtual character playing the role of a person living with dementia in different settings). The 3 scenarios included are as follows:
  - Managing Apathy (Figure S2 in Multimedia Appendix 1): The user will learn strategies to interact with a “mother” character displaying symptoms of apathy.
  - Crisis Response (Figure S3 in Multimedia Appendix 1): The user will respond to and problem-solve a possible fire-hazard situation and interact with a “father” character who may be unaware of the smoking pan in the kitchen.
  - Refusal of Care (Figure S4 in Multimedia Appendix 1): The user will interact with a “father” character who is displaying resistance to care, specifically refusal to get dressed.
3. Feedback and reflection: To reinforce users’ learning after scenario completion, custom feedback is provided by a holographic clinician avatar and simulated person living with dementia within the same scenario environment, based on the particular decisions selected by the user. Immediate feedback and guided reflections from these characters will help caregivers understand the emotional and practical implications of their decisions.

The intervention is intentionally designed to be self-paced and may be repeated to reflect real-world use by caregivers. No minimum exposure dose is specified beyond prespecified adherence thresholds, which are used solely to inform feasibility. Exposure will be operationalized descriptively using engagement metrics including the number of scenarios completed, total time spent in scenarios, and whether virtual clinician feedback was reviewed. These metrics will be summarized to characterize the patterns of use and feasibility. No post hoc exposure-based subgroup analyses or inferential dose-response analyses are planned, since such analyses would risk bias and overinterpretation in a small pilot sample.

### Participants

Participants will be recruited from various partner sites and complete an expression of interest form to self-refer to the study. Partner sites are responsible for sharing our research ethics board (REB)-approved recruitment material for this study. To be eligible to participate, the following inclusion criteria should be met:

- 18 years of age or older.
- Able to provide informed consent.
- Current unpaid family caregivers or care partners of people living with dementia.
- Caring for an individual with either suspected or diagnosed dementia. A formal diagnosis is not required.
- No self-report of active or unstable major psychiatric conditions (eg, major depressive disorders, bipolar disorder, schizophrenia). The study co-principal investigator or joint senior author and geriatric psychiatrist AMB will make the final decisions if necessary.
- Able to read and speak English and able to (1) participate in prototype testing, (2) participate in a postintervention feedback survey, and (3) follow instructions from research staff and complete self-administered surveys or assessment tools.

### Study Enrollment and Timeline

Upon enrollment in the study, participants will complete three key activities (Textbox 1):

1. In-person orientation session: The study team led by primary author (MC) will guide participants one-on-one on how to safely use and take care of a VR-SIM Carers VR headset. A commercially available VR headset (Meta Quest 3), with the VR-SIM Carers software installed, will be loaned to participants for 4 weeks; a user manual and other supplementary materials will also be provided for review.
2. Complete high-fidelity simulation scenarios: Using the VR headset, participants will engage in a series of self-paced immersive scenarios—Managing Apathy, Crisis Response, and Refusal of Care—that represent caregiving situations commonly encountered by family caregivers or care partners of people living with dementia. Participants will review the user manual to guide the completion of the 3 caregiving scenarios (8-10 minutes each) on the VR-SIM Carers headset at their own pace. After completing the scenarios, users will write reflections in the user manual following predefined prompts.
3. Self-assessment tools: Participants will complete a set of 9 brief, self-assessment tools (20-30 minutes) at 3 time points (before, after, and 1 month after completing the VR scenarios). These will be emailed to participants and delivered through SimpleSurvey [23].

Textbox 1. **Timeline or workflow of research activities to be completed by study participants.**

**Week 1: In-person orientation event (90 min)**

- In-person visit at the recruitment site
- Complete REB-approved consent form and baseline assessment scales (via SimpleSurvey)
- Sign out study VR headset; complete orientation RE: headset use led by the study team. Complete Simulator Sickness Questionnaire

**Weeks 2 and 3: Self-paced learning on VR-SIM Carers**

- Self-completion of VR simulation scenarios
- Review virtual clinician’s feedback; program resources
- Complete check-in phone call from the study team

**Week 4: In-person visit by study team (1 h)**

- Complete time 2 assessment scales (via SimpleSurvey)
- Semistructured interview to capture participants’ experience with VR-SIM Carers
- Return study virtual reality headset; submit written reflection notes

**One month after**

- Complete time 3 assessment scales (via SimpleSurvey)
- Provide written feedback on overall study experience (via SimpleSurvey)

### Sample Size

Thirty caregivers will be recruited for the study. A cohort of 5 caregiver participants will be recruited from each recruitment site for equal representation, and enrollment will continue until the targeted number is reached. Recruitment sites are Ontario Shores Centre for Mental Health Sciences in Oshawa, Ontario; Baycrest Centre in Toronto, Ontario; Carefirst Community & Senior Services Association, in Toronto, Ontario; London Health Sciences Centre in London, Ontario; and St. Mary’s Hospital in Montreal, Quebec. Recruitment will take place once local ethics clearance has been received from each site’s research ethics board.

### Power Calculation

Based on previous literature, a minimum sample size of 20 participants has been identified as appropriate for a pilot study to provide preliminary estimates of variability and to evaluate key feasibility parameters. These estimates are informed by existing caregiver pilot study interventions [24,25]. To account for expected attrition, a total sample size of 30 participants will be recruited. This target provides a buffer to maintain the minimum sample needed to evaluate feasibility outcomes and to estimate variance for future sample size calculations, while accounting for potential data loss due to loss to follow-up.

### Progression Criteria

We prespecified quantitative and qualitative progression criteria to guide decisions about whether to proceed to a future definitive trial. Key thresholds are recruitment success 80% or higher (≥24/30 participants) of the target, amend-and-reassess if 50% to 79% (15-23/30) are enrolled, and stop or redesign if less than 50% (<15 participants) are enrolled. Retention success is 80% or higher completion of both postintervention and 1-month follow-up assessments, amend if 60% to 79% (18-23/30) complete, and stop if less than 60% (<18 participants). Intervention adherence success is 75% or more of the participants (≥23/30 participants) completing at least 2 of 3 scenarios and reviewing virtual clinician feedback during the loan period, amend if 50% to 74% (15-22/30), and stop if less than 50% (<15 participants). Data completeness success is 90% or higher (≥27/30) at baseline and 80% or higher (≥24/30) at post-intervention and 1-month follow-up for primary caregiver measures; amend for baseline 75% to 89% (≥23/30) or post-intervention or 1-month 60% to 79% (18-23/30). Safety or tolerability success is 15% or lower (<4 participants) reporting moderate or severe simulator sickness or adverse events attributable to device or software and no serious adverse events, amend if 16% to ‐30% (5-9/30) report moderate symptoms that are remediable, or stop if more than 30% (>9 out of 30 participants) report moderate or severe symptoms or any serious adverse event attributable to the intervention. Acceptability success is 70% or higher (≥21/30 participants) reporting willingness to use the MVP again or recommend it, amend if 50% to 69% (15-21/30), or stop if less than 50% (<15/30). Qualitative signals from interviews, reflection notes, and field notes will be used alongside these thresholds to interpret feasibility. Decision rules are to proceed to a definitive trial without substantive changes if most key criteria meet success thresholds, proceed with specified protocol modifications if criteria fall in the amend range but no major safety signals exist, and stop and redesign if multiple key criteria fall in the stop range or qualitative data reveal fundamental, unresolvable concerns. All progression decisions will be reported and justified in the Results section, and the CONSORT pilot flow diagram will present numbers screened, enrolled, completed at each time point, and the reasons for nonparticipation or dropout.

### Outcome Measures

The primary focus of this pilot is feasibility. Feasibility outcomes that will be reported include recruitment and enrollment rates, consent rate, retention and follow-up rates, adherence to the intervention (eg, time spent in scenarios and number of scenarios completed), data completeness, and adverse events or dropouts related to tolerability. Acceptability and usability outcomes will be assessed through participant responses and standardized instruments. Caregiver outcomes are collected as exploratory measures to describe change over time and to estimate variability for future studies. These caregiver outcome measures, completed at the 3 time points where indicated (Table 1), include:

- Caregiver competence: Caregiver Competence Scale [26], a 4-item scale assessing perceptions of ability to manage caregiving tasks effectively.
- Resilience: Connor-Davidson Resilience Scale [27], a 10-item self-report questionnaire assessing ability to cope with adversity.
- Empathy: Empathy Assessment Scale [28], a 13-item scale evaluating cognitive and affective components relevant to caregiving.
- Stress: Perceived Stress Scale [29], a 10-item scale measuring perceived stress.
- Secondary exploratory measures include behavioral disturbances in people living with dementia (Neuropsychiatric Inventory) [30], depressive symptoms (Center for Epidemiologic Studies Depression Scale) [31], caregiver burden (the Burden Questionnaire) [32], and quality of life (Adult Carer Quality of Life Questionnaire) [33].

VR-SIM Carers is also being evaluated for its feasibility, acceptability, and tolerability on caregiver outcomes, and these will be assessed at multiple points. For technical feasibility, participants will be asked about their ability to carry out the study workflow and the amount of time they engaged with the VR scenarios. Recruitment and retention will be tracked to inform feasibility. For acceptability, participants will be asked about willingness to use the MVP on the expression of interest form and consent form. For tolerability, participants will be asked about any side effects related to hardware or software at the week 1 phone check-in, and the number of dropouts due to adverse events will be tracked.

Participants will also complete evidence-based scales that inform acceptability and tolerability. The 18-item Gaming Use Engagement and Severity Scale [34] assesses user satisfaction, usability, and system quality, while the Igroup Presence Questionnaire [35] measures spatial presence, involvement, and realism in VR environments. The Simulator Sickness Questionnaire documents the type and severity of side effects due to hardware and/or software use.

### Data Analysis

#### Qualitative Data

An inductive thematic analysis will be conducted on qualitative data collected from the week 4 audio-recorded interviews with study participants. Interviews will take 30 to 45 minutes and will be facilitated by SCP, KMC, and EO following a semistructured interview guide (Supplementary file B in Multimedia Appendix 1). Secondary sources of qualitative data will include participant reflection notes, the week 2 or 3 check-in phone call, and the 1-month qualitative written feedback survey questions. This multimodal approach is intended to capture participants’ experiences and perspectives over time. The audio recordings of the semistructured interviews will be transcribed verbatim by research staff and will serve as the primary qualitative data source. Transcripts, reflection notes, and field notes will be imported to Dedoose (9.0.107) for analysis [36].

To create the codebook in Dedoose, 3 (MC, SCP, and KMC) members of the study team trained in coding will independently review 3 interview transcripts to generate an initial set of codes and to collaboratively develop a codebook through consensus [23]. To reduce potential bias, the member who transcribed an audio-recorded interview will not be assigned to code that same transcript. A tracking sheet will be created to manage coding assignments and ensure consistent review.

#### Quantitative data

Descriptive statistics will summarize participants’ demographic characteristics and feasibility metrics. Statistical analysis will be completed using SPSS (version 29; IBM Corp) [37]. Paired 2-tailed *t* tests may be used to assess within-subject changes between specific time points as exploratory analyses. Repeated measures statistics will be used to describe change across the 3 time points. Consistent with CONSORT guidance for pilot and feasibility studies, caregiver outcomes (eg, competence, stress, resilience, empathy) are treated as exploratory, and no causal claims regarding intervention effectiveness will be made. Observed changes over time may reflect nonspecific effects such as time, expectancy, repeated measurement, or general study contact (eg, orientation and check-in calls). Repeated measures are collected to describe the direction and magnitude of change over time and assess the feasibility and sensitivity of the selected measures in this population and delivery format. All quantitative analyses are intended to estimate variability and inform sample size calculations and protocol decisions for a future definitive trial. A 2-sided *P* value of <.05 may be reported for descriptive purposes, but hypothesis testing is not the primary aim of this pilot.

### Study Monitoring

Upon enrollment, each participant will be assigned a unique study number. Any research-related information collected (eg, surveys, scales, and questionnaires) will be labeled and stored using this study number to deidentify personal information and uphold confidentiality. A master list linking participant names to their unique study number will be stored separately on the Ontario Shores secured network, which will be password-protected and accessible only to authorized study personnel. Written documents (eg, consent forms) will be stored in a locked filing cabinet at the Ontario Shores Research and Academics Office, with access restricted to study-approved staff.

Survey and deidentified interview data will be password-protected and accessible only to authorized study personnel. Audio recording files will be deleted once transcribed, and identifying information will be removed. Check-in call field notes will be deidentified as well. All study data will be stored on the Ontario Shores Secured Network Drive and will be aggregated for reporting. All assessment tools and scales will be provided on SimpleSurvey [38], a secure survey platform that will allow each participant easy access to completing surveys at their own pace. The data will remain in SimpleSurvey for 60 days following termination of the account in November 2025 [39]. The data collected through this research study will be stored securely for a 7-year period and will be monitored and reviewed by study investigators to ensure confidentiality protocols are upheld, following REB guidelines and protocol procedures.

### Patient and Public Involvement

As per the guidelines of the VR-CORE framework (VR1 phase), the MVP was shaped through a collaborative and iterative process involving demonstration outreach and knowledge summit activities [40,41]. Initial exposure of the pre-MVP prototype to attendees from these events provided the opportunity for diverse backgrounds (clinicians, researchers, and potential end users) to provide feedback on their interaction. Although this feedback did not contribute to the overall study design, attendees’ feedback provided input regarding usability, technological requirements, clinical relevance, and practical considerations that informed the iterative development of the MVP.

As part of these outreach efforts, a detailed manual was presented to all participants at the knowledge summit to guide them through the MVP’s features and activities and support more structured reflection and feedback. This helped to ensure users had a basic understanding of the prototype’s intended functions and use. Additionally, this project benefited from an advisory board that included knowledge users (Ron Beleno and Carla Velastegui), who provided further review and guidance.

To assess the feasibility of the MVP developed, protocol, and user experience, 3 clinician externs from Ontario Shores Centre for Mental Health Sciences engaged in a mock structured run-through of the protocol. First, an orientation session was conducted; then, externs took the VR headset home for 1 week to experiment with the scenarios and review the manual. Their involvement allowed for the real-time identification of the duration of our set activities and addressed any accessibility or technological concerns about the MVP and orientation flow. Feedback led to improvements in system navigation, onboarding materials, and improved preparedness of the study team for subsequent caregiver cohort orientation sessions.

### Ethical Considerations

This study has received ethical approval from the Ontario Shores Centre for Mental Health Sciences Joint Research Ethics Board (JREB 22‐031-B) and secondary approval from the NRC Research Ethics Board (NRC-REB 2023‐23). All participants will provide written informed consent at the time of the in-person orientation session, prior to taking part in any study-related activities. Participation is voluntary and compensated as approved by the research ethics boards, and all data will be deidentified and stored securely in accordance with institutional data protection guidelines. Any identifying information will be kept separate from research data and accessible only to trained research personnel. Participants will be informed of their right to withdraw from the study at any time without consequence. Study findings will be broadly disseminated through peer-reviewed publications, conference presentations, and reports to stakeholders and funders. Knowledge users’ feedback gathered throughout the pilot study will inform future iterations of the VR-SIM Carers MVP and implementation. In addition, a publicly accessible project website will feature blog posts and community updates to extend our outreach effort. By sharing our findings with both academic and community audiences, we aim to contribute to capacity building, as well as establishing the evidence base for scalable, technology-enabled psychoeducation tools for family caregivers or care partners of people living with dementia.

As aforementioned, local ethics clearance has also been received from each recruitment site’s research ethics board.

## Results

The forthcoming results of this pilot study will provide preliminary evidence on the feasibility, acceptability, tolerability, and preliminary efficacy of the VR-SIM Carers MVP as a psychoeducational tool for family caregivers of people living with dementia. Quantitative results will report on changes in caregiver competence, resilience, empathy, and stress across 3 time points (baseline, postintervention, and 1-month follow-up), as well as secondary outcomes, such as caregiver burden, quality of life, depressive symptoms, and perceptions of behavioral disturbances in people living with dementia. These findings will indicate whether the VR-SIM Carers intervention holds promise in improving psychosocial outcomes for caregivers, while also highlighting areas for refinement prior to larger-scale trials.

Qualitative results, derived from interviews, reflection notes, and written survey responses, will complement the quantitative findings by exploring participants’ lived experiences with the intervention, including perceived benefits, usability, and barriers to engagement. Additional feasibility outcomes, including recruitment and retention rates, adverse events, and system usability scores, will further inform whether the MVP is suitable for broader testing.

Funding for the overall project commenced in September 2022, with funds for the study as described in this protocol paper coming in April 2024. Data collection began in March 2025 with a projected end date of March 2026. As of the submission of the manuscript (December 2025), 21 participants have been enrolled. Analysis of both quantitative and qualitative data is expected to be completed by April 2026. The results will then be published in fall 2026 through academic publications, conference presentations, and community-facing outputs, such as reports and project website updates. These findings will directly inform the next stage of intervention development and scaling.

## Discussion

### Principal Results

The current protocol will be the first to evaluate the feasibility of a simulation-based psychoeducation prototype delivered in an immersive VR environment called “VR-SIM Carers.” The study will first assess feasibility, usability, and educational outcomes of using immersive VR to support family caregivers or care partners of people living with dementia. In addition, this study will explore the preliminary efficacy of VR-SIM Carers in improving psychosocial outcomes such as resilience, empathy, and caregiving competence, measured using standardized assessment tools. The qualitative component of the study will explore users’ experiences with the VR technology and the applicability of psychoeducational content in day-to-day, real-life caregiving encounters.

A key strength of the study is the innovative use of commercially available VR technology to deliver high-fidelity caregiving simulations co-designed with knowledge users, such as family caregivers or care partners of people living with dementia and older adults. The participatory approach used in developing the prototype ensured it was meaningfully co-designed with, and for, caregivers. Users’ feedback was integrated into the design process to ensure content relevance and usability. Importantly, content and technology development without co-design would be unlikely to achieve the same level of relevance, acceptability, or impact, as interventions developed in isolation from knowledge users often fail to address real-world needs and contexts [40,42]. Orientation and technical support may reduce barriers to VR adoption and encourage participation among users who may not be comfortable with technology use. This delivery approach and the self-paced nature of the program enhance accessibility for caregivers, allowing them to learn at a time and pace that suits their individual needs, which improves scalability. Additionally, the MVP for this study was developed according to the VR-CORE framework’s best practices for content development and user-centered design (VR1 phase), actively involving patients and health care providers.

Another strength is the incorporation of both quantitative and qualitative data collection across 3 time points. Quantitative assessments via validated scales (eg, Caregiver Competence Scale, Connor-Davidson Resilience Scale, Empathy Assessment Scale, Perceived Stress Scale) alongside qualitative data obtained from reflection notes, semistructured interviews, and written reflections are expected to yield nuanced insights into outcomes and users’ learning experiences and acceptance of the VR-based intervention.

The findings from this pilot study will provide foundational evidence for the acceptability and feasibility of immersive, simulation–based psychoeducation delivered via VR for family caregivers or care partners of people living with dementia. Should the VR-SIM Carers intervention demonstrate positive outcomes in reducing caregiver stress and improving resilience, empathy, and caregiving competence, future research will focus on large-scale, multisite randomized controlled trials to confirm efficacy and generalizability across diverse caregiver populations and care settings [16]. Further investigation is warranted to identify which caregiver subgroups benefit most, optimal frequency and duration of VR-based interventions, and ways to integrate such technology into existing community and health system supports.

To enhance clinical relevance and impact, future studies of VR-SIM Carers may incorporate the assessment of dementia staging to better target and tailor the intervention for family caregivers or care partners most likely to benefit [43,44]. Longitudinal research will be critical to assess sustained impacts on caregiver well-being, care recipient outcomes, and potential reductions in health system utilization or delayed institutionalization [45]. Additionally, qualitative studies exploring user experience, barriers to adoption, and co-design with caregivers from marginalized or rural communities will be essential to ensure broad accessibility and cultural relevance [16]. Clinically, if VR-SIM Carers proves feasible and acceptable, it could be incorporated as a scalable adjunct to traditional caregiver education, offering flexible, experiential training that complements in-person or online resources. Orientation materials may be adopted into an online Learning Management System to further improve accessibility. Integrating immersive VR simulation into caregiver support programs may ultimately contribute to more sustainable, person-centered dementia care by empowering caregivers with practical skills and emotional coping strategies [12,16].

### Limitations

Several limitations should be acknowledged. The small sample size, although appropriate for a pilot study, limits the statistical power to detect moderate effects and reduces generalizability. For this initial pilot, only English-speaking participants are included, and participants will not be experiencing significant psychological distress, which may exclude some caregivers most in need of support. The pilot’s self-referral recruitment process through the expression of interest form may introduce selection bias, favoring individuals more open to using novel technology. Additionally, variability in participants’ familiarity with VR technology could affect the consistency of the intervention’s delivery. The in-person orientation session, although centrally located, may be a barrier to participation. Finally, individuals in the early stages of dementia typically do not exhibit the behavioral and psychological symptoms of dementia that the VR-SIM Carers MVP is designed to address; as such, the intervention may have limited relevance for caregivers supporting individuals in these early stages [43,44]. These limitations will be carefully considered in interpreting pilot findings and designing future larger-scale studies in diverse caregiver populations (eg, cultural groups) and settings (eg, community vs long-term care).

### Comparison With Prior Work Published in the Literature

Traditional caregiver support programs (in-person or online) have been shown to significantly reduce caregiver strain [46]. Previous VR interventions for caregivers (eg, dementia-simulation programs) demonstrated modest gains in competence [47]. VR-SIM Carers is novel in its integration of evidence-based psychoeducational content into a high-fidelity, self-paced VR simulation. This experiential format goes beyond the didactic approaches of earlier studies, potentially amplifying learning through realistic immersion. The VR-SIM Carers prototype was developed iteratively with input from family caregivers and clinicians, following the VR-CORE framework’s recommendation to engage end users in content design (VR1 phase). Involving caregivers as co-designers enhances relevance and acceptability; indeed, research shows that active patient and caregiver involvement in intervention design improves outcomes and satisfaction. Our participatory approach helps ensure that scenarios and lessons reflect genuine day-to-day challenges faced by caregivers.

Earlier VR-for-caregiver studies often focused on a narrow set of outcomes. For example, a recent meta-analysis found that VR training modestly improved caregiver competence [47]. In comparison, our protocol measures a broad suite of psychosocial outcomes (caregiver stress, resilience, empathy, competence) using validated scales at multiple time points. Coupling these quantitative measures with qualitative interviews and reflections provides richer insights into both the efficacy of the intervention and users’ learning processes. This mixed methods evaluation is more comprehensive than many prior trials, which typically lacked longitudinal follow-up or in-depth user feedback.

### Conclusions

This protocol describes a pilot study to assess a pioneering VR-based intervention that leverages self-paced, immersive simulation to provide psychoeducational content for family caregivers or care partners of people living with dementia. The study is poised to offer valuable insights into the feasibility, acceptability, and preliminary efficacy of such an approach, despite inherent limitations related to sample size and variability in technology acceptance. As immersive technologies become more integrated into caregiver support and skills-building, it is essential to evaluate their real-world effectiveness, usability, and ethical implications. This protocol lays the groundwork for advancing VR-based psychoeducation as a tool to build caregiver competence, resilience, and well-being—an urgent priority in the context of caregiver stress and growing dementia care burden. The outcomes of this pilot study could inform the design and delivery of future VR caregiver interventions and could facilitate building partnerships across the dementia care, technology, and community support sectors. The careful, evidence-based advancement of VR-SIM Carers and similar interventions holds meaningful potential to complement existing resources and address the evolving needs of family caregivers or care partners of people living with dementia in Canada and beyond.

## Supplementary material

10.2196/87107 Multimedia Appendix 1 Supplementary materials: Appendix A: Screenshots of non-player characters and virtual environments in the 3 scenarios; Appendix B: Semi-Structured Interview Guide for post-intervention interview with study participants.

10.2196/87107 Peer Review Report 1 Peer review report by the NRC-CIHR Aging in Place Call 2021 Review Committee, National Research Council/Canadian Institutes of Health Research (Canada).

## Abbreviations

CONSORT: Consolidated Standards of Reporting Trials
MVP: Minimally Viable Prototype
VR: virtual reality
