# Supplementary material for: Supporting Unpaid Caregivers of Persons Living With Dementia: Protocol for a Pilot Feasibility Study to Explore Caregiver Outcomes and Impact of a Co-Designed Simulation–Based Psychoeducation Program in Virtual Reality
Source: JMIR Res Protoc. 2026 Mar 5;15:e87107. doi: 10.2196/87107 (PMC12978973; doi:10.2196/87107)
Supplement: Multimedia Appendix 1 [file resprot-v15-e87107-s001.docx]

**Supplementary file A: Screenshots of non-player characters and virtual environment in the 3 scenarios**


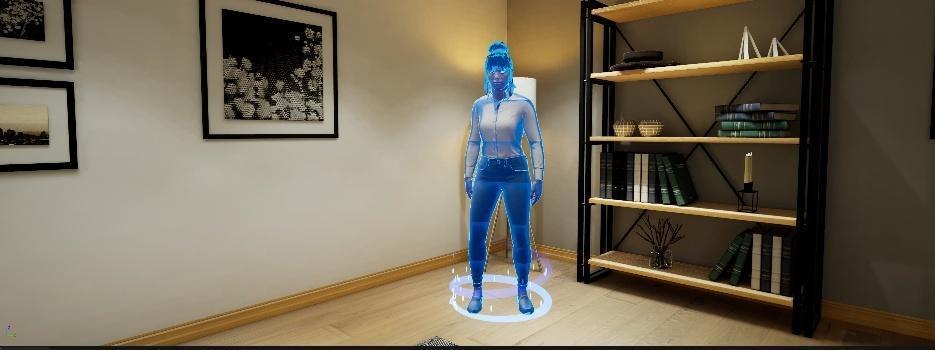


Figure S1: Clinician Avatar “Aiden”

**
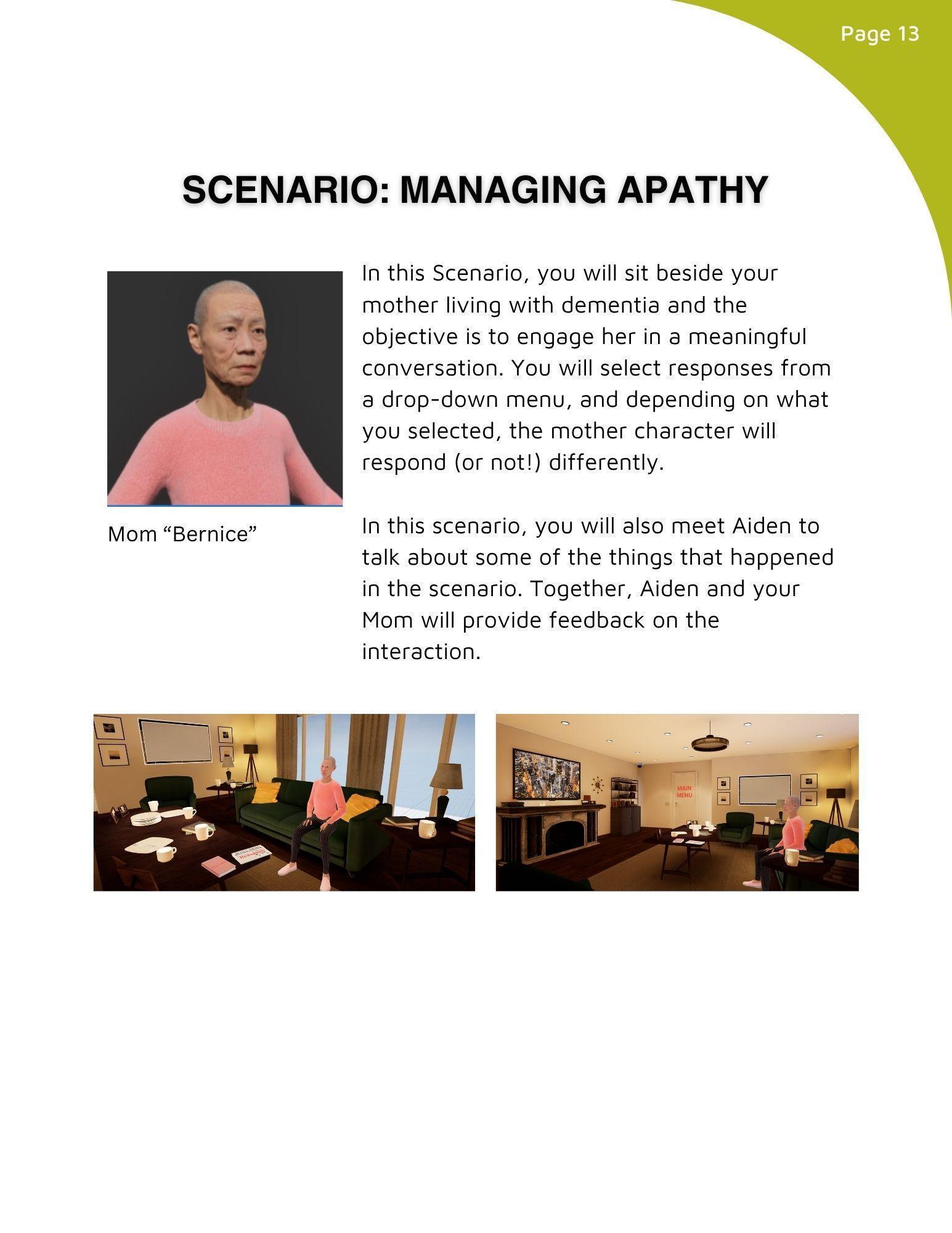
**

Figure S2: Managing Apathy Scenario


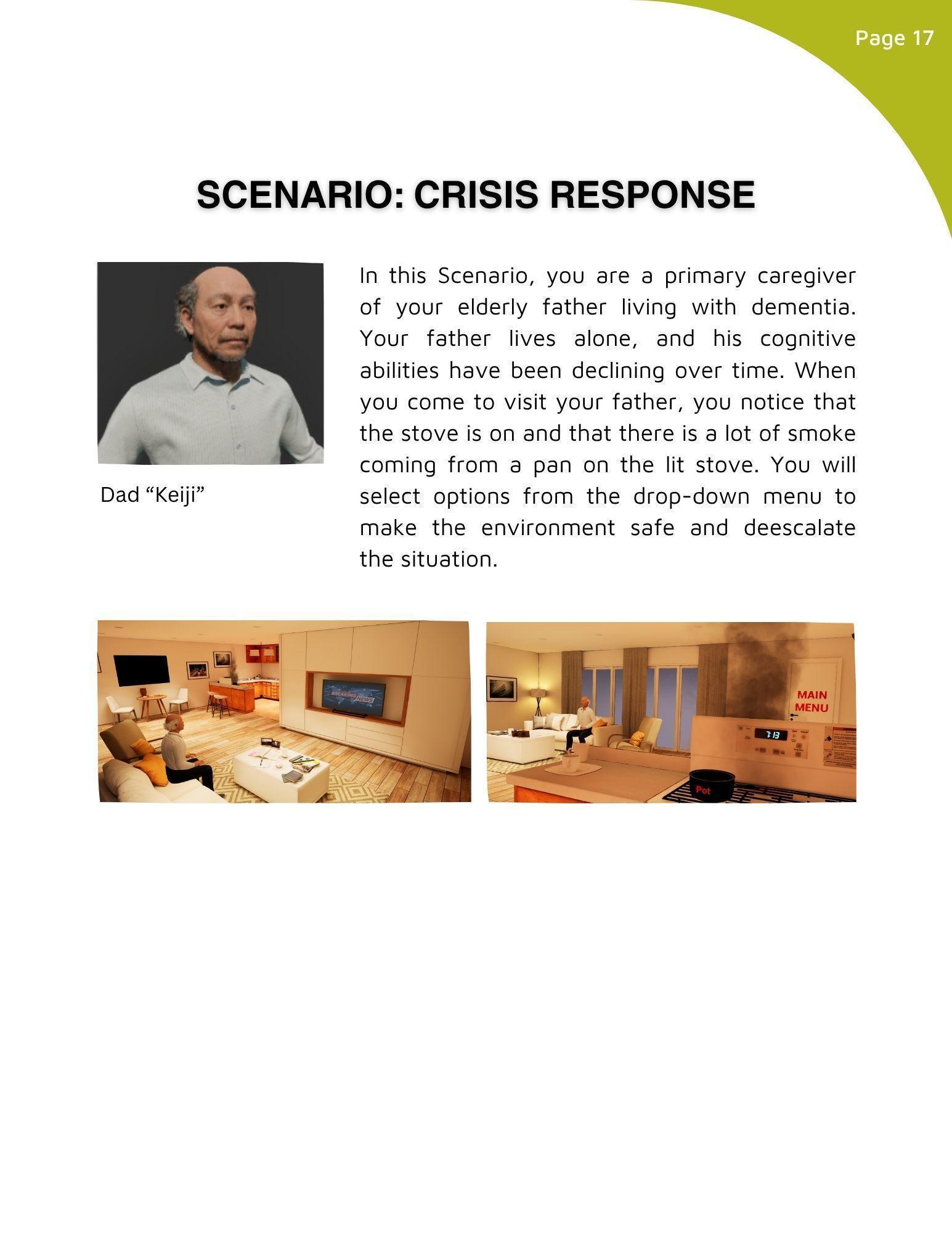


Figure S3: Crisis Response Scenario


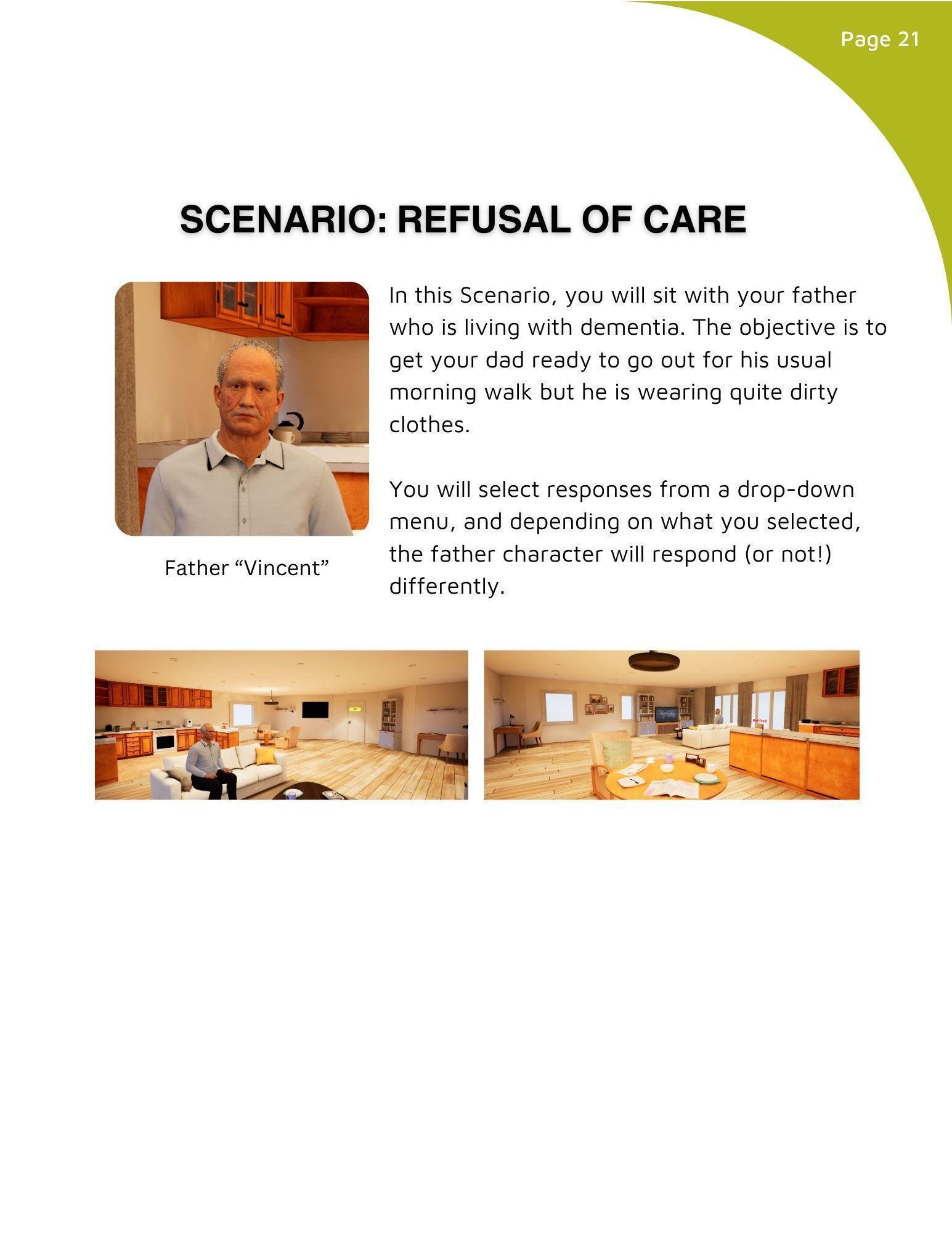


Figure S4: Refusal of Care Scenario

**Supplementary file B: Semi-Structured Interview Guide**

**Post intervention call at Week 4** (Duration: 30 – 45 minutes)

**Objective:** Week 4 reflection; to see what benefits have been gleaned from the program after completing it.

Hello, my name is [insert name] from the VR-SIM Carers Team. Congratulations on completing the program! Today, we’ll be reflecting on your experiences with the VR-SIM Carers scenarios

1. Please comment on your immersive experience:
   1. Did you notice anything different between the 3 scenarios?
      1. Quality of immersion? Narration? Audio? Interaction with the virtual character? Facial expression of virtual character?
2. How did this immersive experience help you understand your role as a caregiver?
3. Have you encountered any situation similar to the ones you saw in the VR-SIM Carers program?
4. How competent or confident would you say you are in managing similar situations in real life?
5. As you walk through the scenarios, what feelings or emotions came to the surface?
   1. Please share any useful strategies you have learned to cope with these feelings or emotions.
   2. Please share any useful strategies you have learned to carry out difficult conversations with the person living with dementia.
6. How likely will you be applying and practicing anything that you have learned into real-life?
7. Is there anything else you’d like to share about your learning experience?

That’s all the questions from my end. Would you have any other concerns or questions currently?

- Yes – answer questions or if unable to answer, tell them you will get back to them.
- No – Talk about return of headset, survey link and honorarium
