# Supplementary material for: Supporting Unpaid Caregivers of Persons Living With Dementia: Protocol for a Pilot Feasibility Study to Explore Caregiver Outcomes and Impact of a Co-Designed Simulation–Based Psychoeducation Program in Virtual Reality
Source: JMIR Res Protoc. 2026 Mar 5;15:e87107. doi: 10.2196/87107 (PMC12978973; doi:10.2196/87107)
Supplement: Peer Review Report 1 [file resprot-v15-e87107-s002.pdf]

NRC·CNRC

PROTECTED A

NRC-CIHR Aging in Place Call 2021
Project Proposal – Reviewer Comments

**Project ID**	AiP-212	
**Project Title**	Dementia Caregivers Skills Training Through Virtual Reality (VR)	
**NPA**	Amer Burhan (Ontario Shores Centre for Mental Health Sciences)	
**NRC PI**	Michael Smith (Medical Devices)	
**Section**	**Reviewer Comments – Strengths**	**Reviewer Comments – Weaknesses**
**Research Quality**	• Theoretical framework linking approach and outcomes are provided.<br>• Recruitment of diverse populations and consideration for intersectionality.<br>• This project has potential to address possible stress points, thereby easing the caregiver load and increasing confidence and encouraging an improved and/or positive relationship with the person with dementia.	• Additional EDI inclusion in the research design (characters & contexts) could strengthen this proposal.<br>• The use of VR specifically is not fully justified.
**Team Composition**	• Strong clinical and knowledge user representation.<br>• Includes implementation and evaluation specialists.<br>• A lot of thought has been put into integration of stakeholders throughout the project.<br>• The team is small, but with various academic collaborators.<br>• The team reflects different disciplines and includes a recent caregiver with technical expertise.	
**Impacts**	• Multiple strategies for knowledge mobilization.	• The knowledge mobilization plan could be more robust with less focus on social media.
**Budget and IP**	• Very good in-kind and student support.<br>• Appropriate distribution with knowledge mobilization activities.	
**Overall Comments**	• Strong team and knowledge user integration.<br>• Likelihood of having an effect is high.	• More EDI consideration could be incorporated into the technology design.
